# Violence Against Women, Mother–Infant Bond and Child Behaviour: An Exploratory Path Analysis at IVAPSA Cohort

**DOI:** 10.1111/cch.70234

**Published:** 2026-02-13

**Authors:** Viviane Costa de Souza Buriol, Marina Nunes, Ariela Raissa Lima‐Costa, Marcelo Zubaran Goldani, Juliana Rombaldi Bernardi, Denise Ruschel Bandeira, Clécio Homrich da Silva

**Affiliations:** ^1^ Saúde da Criança e do Adolescente Universidade Federal do Rio Grande do Sul/UFRGS Porto Alegre Brazil; ^2^ Programa de Pós‐Graduação em Psicologia, da Universidade São Francisco (USF) São Paulo Brazil; ^3^ Serviço de Pediatria do Hospital de Clínicas de Porto Alegre e do Programa de Pós‐Graduação Porto Alegre Brazil; ^4^ Nutrição e Universidade Federal do do Programa de Pós‐Graduação: Saúde da Criança e do Adolescente, Rio Grande do Sul/UFRGS Porto Alegre Brazil; ^5^ Programa de Pós‐Graduação em Psicologia da Universidade Federal do Rio Grande do Sul/UFRGS Porto Alegre Brazil

**Keywords:** child's mental health, maternal stress, mother–infant bonding, postnatal depression, violence against women, Violência contra a mulher, vínculo mãe‐filho, depressão pós‐parto, estresse materno, saúde mental infantil

## Abstract

**Background:**

Violence experienced by women has serious consequences for maternal and child health, generating short‐ and long‐term damage.

**Objective:**

The aim of this study was to evaluate the relation between violence experienced by women and the mother–child bond in the first 6 months after childbirth and on the child's mental health at preschool age, mediated by stress, postpartum depression (PPD), breastfeeding and infant sleep. Participants were recruited from three public hospitals in the city of Porto Alegre (Southern Brazil).

**Methods:**

This study is part of a larger research project with the objective to assess the reflex of different intrauterine environments on the child's health. Five mother–child pair assessment interviews were carried out in order to identify the interrelation between violence and mother–child bond and the influence of this bond on preschool children's mental health, mediated by stress, PPD, breastfeeding and infant sleep, tested using a path analysis model.

**Results:**

From the 295 mother–child pairs analysed, 48.8% of the women experienced violence in their lives and 15.9% during their pregnancy. There was a statistically significant association among violence, stress, PPD, mother–child bond and externalization problems for preschoolers. Violence is correlated with the mother's stress, which is directly linked to the PPD, and thus, indirectly, to the mother–child bond.

**Conclusions:**

This study found that the violence experienced by women before and during pregnancy interferes negatively both in the mother's health and in the mother–child bond, and the weakened mother–child bond can positively explain the externalization problems for preschoolers. Early prevention and identification of the occurrence of violence against women could avoid negative outcomes for the mother–child bond and behavioural problems for preschool‐aged children, as well as in their future life.

## Summary

- The prevalence of violence against women during pregnancy is high.
- Violence is correlated with the mother's stress, which is directly linked to the PPD, and thus, indirectly, to the mother–child bond.
- The violence experienced by women before and during pregnancy interferes negatively both in the mother's health and in the mother–child bond, and the weakened mother–child bond can positively explain the externalization problems for preschoolers.
- Early preventing and identifying the occurrence of violence against women could avoid negative outcomes for the mother–child bond and behavioural problems for preschool‐aged children, as well as in their future life.

## RESUMO

A violência sofrida pela mulher traz graves consequências para a saúde materna e infantil gerando prejuízos de curto e longo prazo.

**Objetivo:**

O objetivo deste estudo foi avaliar a relação entre violência sofrida pela mulher e o vínculo mãe‐filho nos primeiros seis meses após o parto e sobre a saúde mental do pré‐escolar, mediadas pelo estresse, depressão pós‐parto (DPP), aleitamento materno e pelo sono da criança.

**Método:**

Os participantes foram recrutados de três hospitais públicos de Porto Alegre (Brasil). Este estudo faz parte de uma pesquisa maior que teve como objetivo avaliar o reflexo de diferentes ambientes intrauterinos na saúde da criança. Foram realizadas cinco entrevistas com os pares mãe‐filho para identificar a relação entre violência e vínculo mãe‐filho e a influência deste vínculo na saúde mental do pré‐escolar, mediado pelo estresse, DPP, aleitamento materno e sono infantil testado pelo modelo de path analysis.

**Resultado:**

Entre os 295 pares de mãe‐filho analisados, 48,8% das mulheres sofreram violência na vida e 15,9% durante a gravidez. Houve associação estatisticamente positiva entre violência e estresse, DPP, vínculo mãe‐filho e problemas de externalização da criança em idade pré‐escolar. A violência está correlacionada com o estresse materno, que por sua vez está diretamente associado com a DPP e, assim, indiretamente, no vínculo mãe‐filho.

**Conclusões:**

Este estudo constatou que a violência vivenciada por mulheres antes e durante a gravidez interfere negativamente na saúde materna e no vínculo mãe‐filho, e o vínculo mãe‐filho fragilizado pode explicar positivamente os problemas de externalização em pré‐escolares. Prevenir e identificar, precocemente, a ocorrência de violência contra mulheres pode evitar desfechos negativos para o vínculo mãe‐filho e para problemas comportamentais da criança em idade pré‐escolar e no futuro.

## 1 Introduction

Violence against women is a public health problem that affects 25% of those aged 15–24 years of age (ONU 2021). About 30% of women worldwide have already been subjected to physical and/or sexual violence (WHO 2021b). In the poorest countries, it is estimated that 37% of women suffer intimate partner violence. The highest rates can be found in Oceania, South Asia and sub‐Saharan Africa (33% to 51%), while the lowest rates (16%–23%) are in Europe (WHO 2021a).

In Brazil, in 2019, 7.60% of 18‐ to 59‐year‐old women experienced intimate partner violence, with a higher prevalence among the youngest and poorest (de Vasconcelos et al. 2021); during the COVID‐19 pandemic, the number of cases increased globally. Stress stemming from the fear of illness, social isolation and prolonged cohabitation with the partner exacerbated the precipitating factors of intimate partner violence. There was an increase mainly in moral, verbal and psychological violence when compared to previous years (Moreira and Pinto da Costa 2020; Lausi et al. 2021; Rhodes et al. 2020; Smyth et al. 2021).

According to the Brazilian Forum on Public Security (2022), between March 2020 and December 2021, 2451 cases of uxoricide and more than one hundred thousand cases of rape of female victims were reported (Brazilian Forum on Public Security 2022), with an estimated loss of one billion Brazilian reais of absenteeism in the labour market and the perpetuation of poverty motivated by domestic violence in Brazil (USP/AUN 2019).

Violence against women has far‐reaching repercussions. It limits the political and economic participation of women, increases the demand for health care services and also overburdens areas such as care, education and justice (Netto et al. 2014). This violence is not only limited to immediate physical damage but also has long‐term repercussions such as depression, suicide attempts and unwanted pregnancy (Islam et al. 2014).

Pregnancy, contrary to what is expected, does not inhibit violence. There is a great variation in existing prevalence figures: no less than 1 in 50, and every 1 in 2 women may be experiencing physical IPV during pregnancy (Román‐Gálvez et al. 2021). Violence experienced during pregnancy reflects on the mother–child bond and the child's mental health in early childhood (McIntosh et al. 2021; Silva et al. 2018). Violence experienced in childhood, or physical, sexual or psychological abuse practiced by an intimate partner, is associated with postpartum depression (Zhang et al. 2019).

It is known that the emotional condition of the mother, such as anxiety, stress and postpartum depression, plays a major role in the pattern of breastfeeding (Gila‐Díaz et al. 2020), the mother–child bond (Lutkiewicz et al. 2020) and the child's neurodevelopment (Toso et al. 2020). Breastfeeding, as a moment of exchange between the mother and her baby, discloses an important time for both the infant and the mother. The hormonal processes associated with breastfeeding protect mothers from postpartum depression by attenuating the response to cortisol by stress, besides regulating sleep and wakefulness patterns, improving the mother's emotional involvement with the child and reducing temper difficulties (Figueiredo et al. 2013). Violence during pregnancy is associated with an unfavourable breastfeeding practice, presenting a lower chance of breastfeeding intention, early interruption of exclusive breastfeeding, lower chance of starting breastfeeding and short duration (Mezzavilla et al. 2018).

Women suffering from postpartum depression usually breastfeed for a shorter period (Dias and Figueiredo 2015). The incidence of depression in the first 6 months postpartum affects the safety of the child's bond (Śliwerski et al. 2020), while maternal stress and anxiety levels contribute to negative outcomes in the mother–child interaction as well as the child's development (Lutkiewicz et al. 2020; Tichelman et al. 2019). Parental depression also influences infant sleep patterns (El‐Sheikh and Kelly 2017), and it is associated with the mother's perception of the existence of difficulties in the baby's sleeping. The mother's own sleeping difficulties correlate with the baby's sleep maintenance problems (Rico and Sá 2015). Bond safety is associated with greater sleep efficiency, while resistance to the child attachment is exclusively—and more strongly—associated with sleeping problems (Simard et al. 2017).

The correlation of violence to the mother's and child's mental health and mother–child bond has been an object of investigation (Zhang et al. 2019). Souza et al. (2024) observed that impaired mother–infant bonding was associated with less maternal schooling, poor bonding with their mothers during childhood, violence and postpartum maternal depression. Nevertheless, few studies evaluate the role of lifetime and prenatal violence as risk factors regarding postpartum emotional problems for children (Tien et al. 2020).

### 1.1 Objectives and Hypotheses of the Study

The study aimed to evaluate the association of violence experienced by mothers with the mother–child bond during the first 6 months postpartum and on preschoolers' mental health, examining the mediating factors of stress, postpartum depression, breastfeeding and infant sleep. We hypothesize that violence affects the mother–child bond, subsequently influencing preschoolers' mental health.

## 2 Methods

### 2.1 Design and Site

This longitudinal study is part of a research entitled the ‘Impact of Perinatal Different Intrauterine Environments on Child Growth and Development in the First Six Months of Life ‐ IVAPSA study’. Details about the convenience sample size and protocols used in this study have been published elsewhere (Bernardi et al. 2012), as well as some baseline results (Werlang et al. 2019).

The postpartum recruitment and interviews occurred between 2011 and 2019. The study included 400 mother–infant pairs whose delivery assistance took place in three public hospitals of reference for the Brazilian National Health System. The mother–child pairs were distributed into five interest groups of study, according to the following maternal gestational conditions: hypertension, diabetes mellitus, smoking, intrauterine growth restriction (IUGR) with the birth of SGA newborns (small for gestational age) and a control group. Exclusion criteria were HIV‐positive newborns, twins or higher‐order multiples, preterm (gestational age under 37 weeks), those with congenital malformations or those requiring hospitalization.

To avoid the influence of other maternal metabolic factors during pregnancy, mother–child pairs with hypertension, diabetes mellitus and smoking groups were not used. The sample is a convenience sample, and participants with maternal hypertension, diabetes and smoking were excluded to avoid confounding metabolic conditions. Thus, according to the objectives of this study, two groups were used: (1) SGA group: newborns at term with IUGR who had a birth weight below the 5th percentile for fetal growth (Alexander et al. 1996) and (2) non‐SGA group: newborns at term without IUGR. For both groups, there were no mothers with any of the gestational conditions investigated by the IVAPSA research (hypertensive disorders, diabetes mellitus or smoking during pregnancy).

### 2.2 Data Collection

According to the planned research protocol, after the initial interview in the first 48‐h postpartum, the interviews and evaluations were scheduled for the 7th and 15th days, for the 1st, 3rd and 6th months, and from 3 to 5 years to monitor the child's health periodically. For this study, information obtained in the postpartum period and at the 1st, 3rd and 6th months and children aged 3–5 were used.

### 2.3 Participants

The sample of the present study was composed of 295 mother–child pairs, who answered the questionnaire on violence. Five of them did not want to answer the questionnaire, and there was a loss of segment for another 100 (26 due to refusal to continue participating in the research, and the others, the address could not be found). To compare the representativeness of the subsample among the women who answered the violence questionnaire, based on the study of this article with the original sample, a Chi‐square analysis was performed. From them, only the mother's age, marital status and the partner's work situation presented any difference in values: older women (> 30 years, 34.2% vs. 21.0%, *p* = 0.037), women who live with a partner (82.0% vs. 72.4%, *p* = 0.050) and the ones whose partner has a signed work permit (65.1% vs. 55.2%, *p* = 0.005) are those who prevailed more in the questionnaire on violence.

### 2.4 Procedure and Variables

Eligible participants were recruited and interviewed 24 to 48 h after delivery by checking the medical records of the women and newborns' postpartum data from hospitals included in the study. Once the mothers agreed to participate, they were invited to sign the Informed Consent Form and receive reminders of their subsequent interviews. An identification number has been assigned to each pair, ensuring the data confidentiality. The information was extracted from the medical record, the child's birth certificate and the first interview in the hospital, when the socioeconomic classification questionnaire was also applied. The follow‐up was carried out in the 1st, 3rd and 6th months of the infant's life and, later, between 3 and 5 years of the child's life (Table 1).

**TABLE 1:** Collection logistics—IVAPSA cohort, Porto Alegre, Brazil.

Interview	Stages of the interview	Location	Variables/instrument
1st	Postpartum	Rooming‐in care	Informed Consent Form (ICF) Socioeconomic information (ABEP) Sociodemographic, pre and perinatal variables/postpartum questionnaire
2nd	1 month	CPC	Violence (ASS) Perceived stress/PSS‐14
3rd	3 months	Domicile	Postpartum depression (EPDS)
4th	6 months	CPC	Violence (ASS) Postpartum depression (EPDS) Infant sleep (SBQ) Breastfeeding and family behaviours and routine/follow‐up questionnaire of 6 months Mother–child bond (PBQ)
5th	3–5 years of the child	CPC	Child's mental health (SDQ)

*Note:* Abuse Assessment Screen (Violence), Perceived Stress Scale (PSS), Edinburgh Postnatal Depression Scale (EPDS), Sleep Behaviour Questionnaire (Sleep), Breastfeeding (AM), Postpartum Bonding Instrument (PBQ), Strengths and Difficulties Questionnaire (SDQ).

Abbreviation: CPC, Clinical Research Center of Hospital de Clínicas de Porto Alegre.

For this study, the following variables were evaluated: (a) sociodemographic: social class, father's age and schooling, father's work situation and number of adults in the house; (b) behaviour and family routine: alcohol intake by the father or partner, if the child goes to daycare at 6 months, if the mother takes care of the child at 6 months and if the mother used to read to the child; (c) maternal and obstetric: age, schooling, work situation, mother lives with partner, number of children, whether pregnancy was planned, use of drugs in pregnancy, violence, stress, postpartum depression; (d) care variables: number of prenatal appointments and type of delivery; (e) of the newborn: weight (g), head circumference (cm), Apgar index and gestational age; (f) of mother–child relationship: breastfeeding at 6 months, mother–child bond; and (g) the child at preschool age: mental health.

### 2.5 Evaluation Instruments Used

#### 2.5.1 Sociodemographic and Clinical Questionnaire

Sociodemographic measures: In the first interview, and through consultation of the hospital records, information on the mother's age, parity, type of delivery (vaginal or caesarean), number of prenatal appointments, use of drugs in pregnancy, whether pregnancy was planned or not, marital status, educational level and work situation was collected. Regarding the father's information, age, schooling, work situation and alcohol intake were collected. Gestational age and head circumference, Apgar 1' and 5', sex and birth weight of newborns were also included. If the child attends daycare, the presence or absence of breastfeeding was also investigated.

#### 2.5.2 Socioeconomic Classification

For a socioeconomic evaluation, an instrument used by the government to classify the Brazilian population into categories A, B, C, D and E was applied. Among them, category A was considered the richest socioeconomic class, and category E the poorest. This instrument considers the level of education, consumer goods, purchase power and number of specific rooms in the house. Maternal skin colour was defined by the interviewer as white or non‐white.

#### 2.5.3 Questionnaire Addressing Violence

The violence experienced by women in life and violence during pregnancy was evaluated using the *Abuse Assessment Screen* self‐report questionnaire developed by the international team of the WHO multicountry study on women's health and domestic violence. A Portuguese version validated by Reichenheim et al. (2000) was applied in the privacy of a reserved room in the 1st and 6th months after the delivery. For this study, two variables were created with single Yes or No answers. The first, named ‘Total Violence’ (VT), considers affirmative answers for the questionnaires on the 1st and 6th months for these four questions: (a) ‘Have you ever been humiliated?’; (b) ‘Have you ever been assaulted?’; (c) ‘Have you ever been attacked?’; and (d) ‘Have you ever been forced to have sex?’. And the second, named ‘Violence in Pregnancy’ (VP), for affirmative answers to these other four questions: (a) ‘Were you humiliated in pregnancy?’; (b) ‘Were you assaulted in pregnancy?’; (c) ‘Were you attacked in pregnancy?’; and (d) ‘Were you forced to have sex in pregnancy?’ The affirmative answer to one or more questions classified the woman as a victim of violence.

#### 2.5.4 Postpartum Bonding Questionnaire (PBQ)

The interaction between the mother–child bond was evaluated by a specific questionnaire applied in the children's 6 months of age. The questionnaire is self‐applicable and the version used was the Portuguese version of ‘The Postpartum Bonding Instrument’ (PBQ) (Baldisserotto et al. 2018; Nazaré et al. 2013), translated from the original questionnaire (Brockington et al. 2001). The protocol consists of 25 items, with answers varying from always = 0; 1 = *very often*; 2 = *often*; 3 = *sometimes*; 4 = *rarely* and 5 = *never*; and four subscales: (1) ‘impaired mother‐infant bonding’, formed by 12 items; (2) ‘rejection and anger’ (seven items); (3) ‘anxiety about care’ (four items) and (4) ‘risk of abuse’ (two items) (Brockington et al. 2006; Hernández et al. 2015). Currently, this is the most used instrument to diagnose problems in the mother–child relationship, and the subscale 1, weakened bond, is sensitive to identify mothers with minor dysfunction in the mother–child bond, which is relevant to our study (Perrelli et al. 2014). Here, we used only the first subscale, composed of 12 items, to evaluate the weakened bond between mother and child, in which, due to its significance, the higher the score of the mother the weaker the bond is. In the present study, Cronbach's alpha index was 0.70.

#### 2.5.5 Perceived Stress Scale—PSS 14

To evaluate the perceived stress level of the mother, the Perceived Stress Scale PSS 14, translated and validated by Luft et al. (2007), was used (Cohen et al. 1988). The scale was applied in the first month after delivery. It consists of 14 items with answers ranging from zero to four (0 = *never*; 1 = *almost never*; 2 = *sometimes*; 3 = *almost always*; 4 = *always*). Questions with positive connotation (4, 5, 6, 7, 9, 10 and 13) have their score added inverted. The other questions are negative and should be added directly. The sum of the score of the questions provides scores that can range from zero (without stress) to 56 (extreme stress). Values lower than or equal to 30 represented the absence of symptoms for maternal stress, and higher than this, its presence (Cohen et al. 1988). In the present study, Cronbach's alpha index was 0.85.

#### 2.5.6 Edinburgh Postnatal Depression Scale (EPDS)

Postpartum depression was evaluated using the EPDS, which was adapted to the Brazilian population by dos Santos et al. (1999), and used in the 3rd and 6th months of the child's life. The one with the highest score was used for the purpose of this work, as we were interested to know if the woman presented PPD up to 6 months after childbirth, and not all women answered the EPDS at both moments of collection. EPDS is a self‐report instrument composed of 10 items, referring to the last 7 days, whose options are scored (0, 1, 2 or 3), according to the presence or intensity of the symptom. Its items include psychic symptoms, such as depressive mood (feeling of sadness, self‐devaluation and feeling of guilt, ideas of death or suicide), loss of pleasure in previously considered enjoyable activities, fatigue, decreased ability to think, concentrate or make decisions, as well as physiological symptoms (insomnia or hypersomnia) and behavioural changes (crying crises). The sum of the points makes a score of 30, being considered as depressive symptomatology equal to or greater than 12, as defined in the adaptation of the scale for the Brazilian sample (dos Santos et al. 1999). In the present study, Cronbach's alpha index at 3 months was 0.85 and 0.83, applied at 6 months.

#### 2.5.7 Sleep Behaviour Questionnaire (SBQ)

In relation to infant sleep, quantitative parameters were explored through two questions: (1) What is the total amount of the child's sleep during the night? and (2) What is the child's total sleep duration in 24 h? The latter is obtained through the sum of the number of hours of sleep during the day and the night. Those questions are included in the SBQ (Cortesi et al. 1999), translated and adapted to Portuguese (Batista and Nunes 2006), which was then adapted to the context of this study. It was applied to the mother, or main caregiver, at the sixth month of the child, aiming to reflect her regular sleep characteristics at this age.

#### 2.5.8 Strengths and Difficulties Questionnaire (SDQ)

To track the child's mental health problems, the SDQ (Goodman 1997) scale was used, translated and validated in Brazil by Fleitlich et al. (2000). This instrument is used for children and adolescents 2–16 years of age, containing 25 questions and five subscales (prosocial behaviour, hyperactivity/attention deficit, emotional, conduct and relationship problems). The answer options are false, more or less true or true. The sum for each subscale, as well as the total values, allows the classification of the individual into three categories: normal behaviour (0–15 points), borderline behaviour (16–19 points) and deviant behaviour (20–40 points). In almost all subscales (except prosocial behaviour), the higher the score, the greater the number of symptoms. For this research, we used a combination of three subscales, which compounds ‘emotional symptoms’ and ‘peer relationship problems’ an ‘internalization problems’ subscale (anxiety, depression, shyness and inferiority complex), and the subscales for ‘behavior problems’ and ‘hyperactivity’ combined into ‘externalization problems’ subscale (refleting challenging behaviour, impulsivity, aggression and hyperactivity), and a the ‘prosocial’ subscale was retained as a separate measure (Goodman et al. 2010). In the present study, Cronbach's alpha index for Prosocial subscale was 0.32, Externalizing subscale was 0.75 and Internalizing subscale was 0.70.

### 2.6 Statistical Analysis

After checking the consistency of the final database, a descriptive analysis of the continuous and categorical variables was conducted. Comparisons of sociodemographic characteristics, family behaviour and routine, maternal and obstetric factors, care practices, and newborn between the group that experienced violence and the group that did not were performed. Parametric variables were expressed as mean (± standart deviation), and analyzed using Student's *t*‐test, while nonparametric variables were presented as median and interquartile range and analyzed using the Mann‐Whitney test. The Chi‐square test was performed to detect differences among categorical variables (maternal age, maternal and paternal schooling, social class, mother and father work with a signed work permit, if pregnancy was planned, if the mother lives with a partner, number of children, sex of the child, type of delivery, use of drugs during pregnancy, alcohol intake by the partner, if the child goes to the daycare, if the mother is the one who takes care of the child most of the time and the presence of breastfeeding) followed by the proportion comparison test with the Bonferroni Adjustment for categorical variables, considering a significance level of 5% (*p* < 0.05) and a confidence interval of 95%. Data were analysed using the statistical programme SPSS (Statistical Package for the Social Sciences) version 18.0, as it is the one available in the hospital where the study was conducted.

**FIGURE 1:**
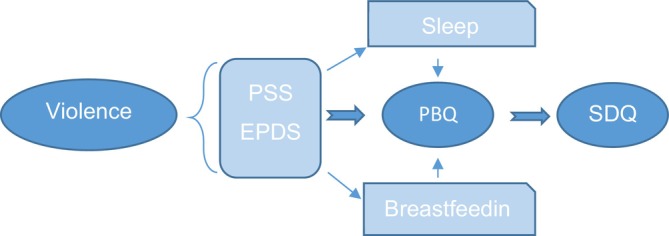
Predefined theoretical model for *path analysis*. Legend: Violence: Abuse Assessment Screen, PSS: Perceived Stress Scale, EPDS: Edinburgh Postnatal Depression Scale, Sleep: Sleep Behaviour Questionnaire, Breastfeeding, PBQ: Postpartum Bonding Instrument, SDQ: Strengths and Difficulties Questionnaire.

Finally, an exploratory path analysis that tested the outcomes of a predefined theoretical model, from which we derived our key results, followed by a diagram with hypotheses relationships based on the literature and the results of the bivariate analysis, was performed (Figure 1). This analysis is an extension of multiple regressions, in which the relations among the variables are expressed in terms of correlation coefficients. The technique itself does not allow us to assume a causality relation, since the directionality among the variables was defined by the authors, but it reflects theoretical models that serve as the basis for the researcher to evaluate which hypothesis best fits the pattern of correlations among the studied variables. Standardized path coefficients (β), unstandardized estimates (B), standard errors and bootstrapped 95% confidence intervals were obtained using 1000 bootstrap samples. Model fit was evaluated using multiple indices, including the Comparative Fit Index (CFI), Tucker–Lewis Index (TLI), Root Mean Square Error of Approximation (RMSEA) with 90% confidence interval, chi‐square test of model fit (χ^2^) and the Standardized Root Mean Square Residual (SRMR). Following conventional guidelines, CFI and TLI values ≥ 0.90, RMSEA values ≤ 0.08 and SRMR values ≤ 0.08 were interpreted as indicative of acceptable model fit (Brown 2006). In the model tested in this analysis, the variables of the mother were considered as mediators: (a) situation of violence, (b) stress (1‐month postpartum), (c) depressive symptoms (3‐ or 6‐month postpartum) and (d) mother–child bond (6‐month postpartum); and the child's variables: (a) amount of sleep and breastfeeding (6 months of life) and (b) child mental health problems (assessed between 3 and 5 years of age). We used only the observed variable, and the analysis was performed in the software Mplus 7th release. Because the variable ‘violence’ was essential to the model, participants missing this information (*n* = 29) were excluded. This may introduce some risk of nonrandom missingness, although the number of excluded cases was small, reducing the likelihood of biasing the findings.

### 2.7 Ethical Aspects

The Ethics and Research Committee of Hospital de Clínicas de Porto Alegre (HCPA) (Protocol 11/0097) and the Grupo Hospitalar Conceição (GHC) (Protocol 11/027) approved the IVAPSA Cohort and, therefore, the present study. All participants were recruited after signing the Informed Consent Form. The project was carried out in accordance with the latest guidelines and current norms of the National Health Council of the Brazilian Ministry of Health (resolutions number 466/2012 and 580/2018). During the interviews, when the health conditions of the participants were an object of concern, they were referred to the reference services.

## 3 Results

The prevalence of violence in life, which we call total violence (VT), was 48.8%, and for women who experienced violence in pregnancy (VP), 15.9% (Table 2). Women with a statistically significant association for VT (144) and for VP (47) were significantly more likely to have higher scores of stress, depression, weakened mother bond and problems in the child's internalization at preschool age. Children whose mothers experienced violence in life had a higher total sleep duration when compared to those who did not experience violence (Table 2).

**TABLE 2:** Characteristics of the sample and main variables for the occurrence or not of violence in life and pregnancy (*n* = 295).

Violence in life (VT)				Violence in pregnancy (VP)		
	Negative	Positive	*p*	Negative	Positive	*p*
	*n* (%)	*n* (%)		*n* (%)	*n* (%)	
**Descriptive variables**						
Social class			**0.003**			0.054
A + B	71 (47.7)^**A**^	42 (29.8)^**B**^		103 (41.9)	10 (22.7)	
C	73 (49.0)^**A**^	86 (61.0)^**B**^		128 (52.0)	31 (70.5)	
D + E	5 (3.4)^**A**^	13 (9.2)^**B**^		15 (6.1)	3 (6.8)	
Father w/signed work permit	104 (68.9)	88 (61.1)	0.308	164 (66.1)	28 (59.6)	0.516
Paternal schooling						
< 8 years	23 (15.2)	34 (23.6)	0.109	42 (16.9)^**A**^	15 (31.9)^**B**^	**0.009**
8 to 11 years	108 (71.5)	87 (60.4)		173 (69.8)^**A**^	22 (46.8)^**B**^	
> 12 years	20 (13.2)	23 (16.0)		33 (13.3)^**A**^	10 (21.3)^**A**^	
Alcohol intake by partner	73 (57.5)	82 (65.1)	0.215	126 (59.2)	29 (72.5)	0.158
Day care at 6 months	10 (8.6)	17 (14.7)	0.152	20 (10.2)	7 (19.4)	0.191
Mother takes care of the child	90 (77.6)	94 (81.0)	0.517	158 (80.6)	26 (72.2)	0.253
Maternal age						
< 20 years	28 (18.5)	23 (16.0)	0.259	40 (16.1)	11 (23.4)	0.446
20 to 29 years	78 (51.7)	65 (45.1)		123 (49.6)	20 (42.6)	
> 30 years	45 (29.8)	56 (38.9)		85 (34.3)	16 (34.0)	
Maternal schooling						
< 8 years	28 (18.5)	41 (28.5)	0.113	52 (21.0)^**A**^	17 (36.2)^**B**^	**0.035**
8 to 11 years	103 (68.2)	89 (61.8)		164 (66.1)^**A**^	28 (59.6)^**A**^	
> 12 years	20 (13.2)	14 (9.7)		32 (12.9)^**A**^	2 (4.3)^**A**^	
Mother w/signed work permit	56 (37.1)	47 (32.6)	0.497	89 (35.9)	14 (29.8)	0.421
With partner	129 (85.4)	113 (78.5)	0.12	210 (84.7)^**A**^	32 (68.1)^**B**^	**0.007**
Number of children			0.452			0.884
Current only	63 (41.7)	50 (34.7)		95 (38.3)	18 (38.3)	
Current +1	12 (7.9)	14 (9.7)		21 (8.5)	5 (10.6)	
3 or +	76 (50.3)	80 (55.6)		132 (53.2)	24 (51.1)	
Planned pregnancy	64 (42.4)	49 (34.0)	0.175	100 (40.3)	13 (27.7)	0.102
Drugs in pregnancy	1 (0.8)	2 (1.5)	0.557	1 (0.5)	2 (4.7)	0.111
Vaginal delivery	98 (64.9)	87 (60.4)	0.426	158 (63.7)	27 (57.4)	0.416
Sex of child female	76 (50.3)	83 (57.6)	0.208	133 (53.6)	26 (55.3)	0.831
Breastfeeding at 6 months^c^	79 (68.1)	82 (70.7)	0.669	137 (69.9)	24 (66.7)	0.849
**× (±SD)/md (27–75)**						
Paternal age^a^	29.2 (7.5)	31.3 (9.8)	**0.038**	30.4 (8.8)	29.4 (8.4)	0.489
Number of adults in the house^b^	2 (2–3)	2 (2–3)	0.359	2 (2–3)	2 (2–3)	0.874
No. of prenatal appointments^b^	9 (6–10)	8 (6–11)	0.779	8 (6–10)	9 (5–11)	0.763
Gestational age DUM^a^	39.0 (2.20)	39.1 (1.49)	0.686	39.0 (1.9)	38.9 (1.7)	0.759
Child birth weight^a^	3219.5 (478.2)	3227.2 (520.0)	0.895	3255.2 (494.2)	3055.5 (490.8)	**0.012**
Child head circumference^a^	33.8 (1.5)	33.9 (1.6)	0.762	33.9 (1.5)	33.3 (1.6)	**0.015**
Child Apgar 1'^b^	9 (8–9)	9 (8–9)	0.763	9 (8–9)	9 (8–9)	0.984
Child Apgar 5'^b^	9 (9–10)	10 (9–10)	0.556	10 (9–10)	10 (9–10)	0.795
**Protocols**						
Mother–child bond—PBQ (factor 1)^a^	2 (1–4)	5 (2–7)	**< 0.001**	3 (1–5)	6 (3–7)	**0.002**
Maternal stress—PSS^b^	16.5 (8.2)	21.3 (8.1)	**< 0.001**	18.0 (8.0)	23.5 (9.3)	**< 0.001**
Maternal depression—EPDS^b^	4.5 (4.5)	7.5 (5.0)	**< 0.001**	5.4 (4.5)	9.4 (5.9)	**< 0.001**
Sleep duration in hours—SBQ^a^	11.3 (2.1)	11.9 (2.4)	**0.043**	11.6 (2.3)	11.7 (2.3)	0.747
Sleep total duration at night in hours—SBQ^a^	15 (10–30)	20 (10–30)	0.171	20 (10–30)	20 (10–30)	0.543
SDQ^b^						
Prosocial problems	8.8 (1.1)	8.6 (1.4)	0.459	8.8 (1.3)	8.5 (1.4)	0.456
Externalizing problems	8.6 (4.7)	9.9 (4.1)	0.150	9.2 (4.4)	9.7 (4.5)	0.703
Internalizing problems	4.2 (2.9)	6.5 (4.0)	**0.004**	5.1 (3.5)	7.0 (4.2)	**0.064**

*Note:* Chi‐square test followed by Z test for proportion with Bonferroni adjustment for categorical variables and *t* or Mann–Whitney test for continuous variables according to distribution (the categories that have an indication of letter A are the ones that presented the highest percentage compared to the group that did not suffer violence. Letter B is the one with the lowest percentage). Overwrite letters indicate the result of multiple comparison (Z test for proportion with Bonferroni adjustment), that is, when the letters are the same, there is no statistically significant differences (*p* < 0.05) among the same categories when compared to the groups with or without the factor of violence. Values in bold present the significant results or threshold *p* < 0.10.

Abbreviations: DUM, Date of last period; EPDS, Edinburgh Postnatal Depression Scale; PBQ, Postpartum Bonding Instrument; PSS, Perceived Stress Scale; SBQ, Sleep Behaviour Questionnaire; SDQ, Strengths and Difficulties Questionnaire.

^a^ Values for the median and the interquartile range when compared to the Mann Whitney test.

^b^ Values presented are median and standard deviation when compared to the *t* student test.

^c^ Values are absolute and relative frequency when compared to the Chi‐square test.

Among the sociodemographic variables (Table 2):

- Women positive for VT belonged most frequently to social classes C, D and E (*p* = 0.003).
- The paternal age was higher among women positive for VT (*p* = 0.038).
- VP was significantly associated with lower maternal education level (< 8 years of study, *p* = 0.035), with extremes of paternal schooling (< 8 and > 12 years of study, *p* = 0.009) and women without a partner (*p* = 0.007).
- Children of women who suffered VP had an average birthweight less than 200 g (*p* = 0.012).
- Head circumference in children of women who experienced VP was, on average, 0.1 cm lower (*p* = 0.015).

### 3.1 Path Analysis

**FIGURE 2:**
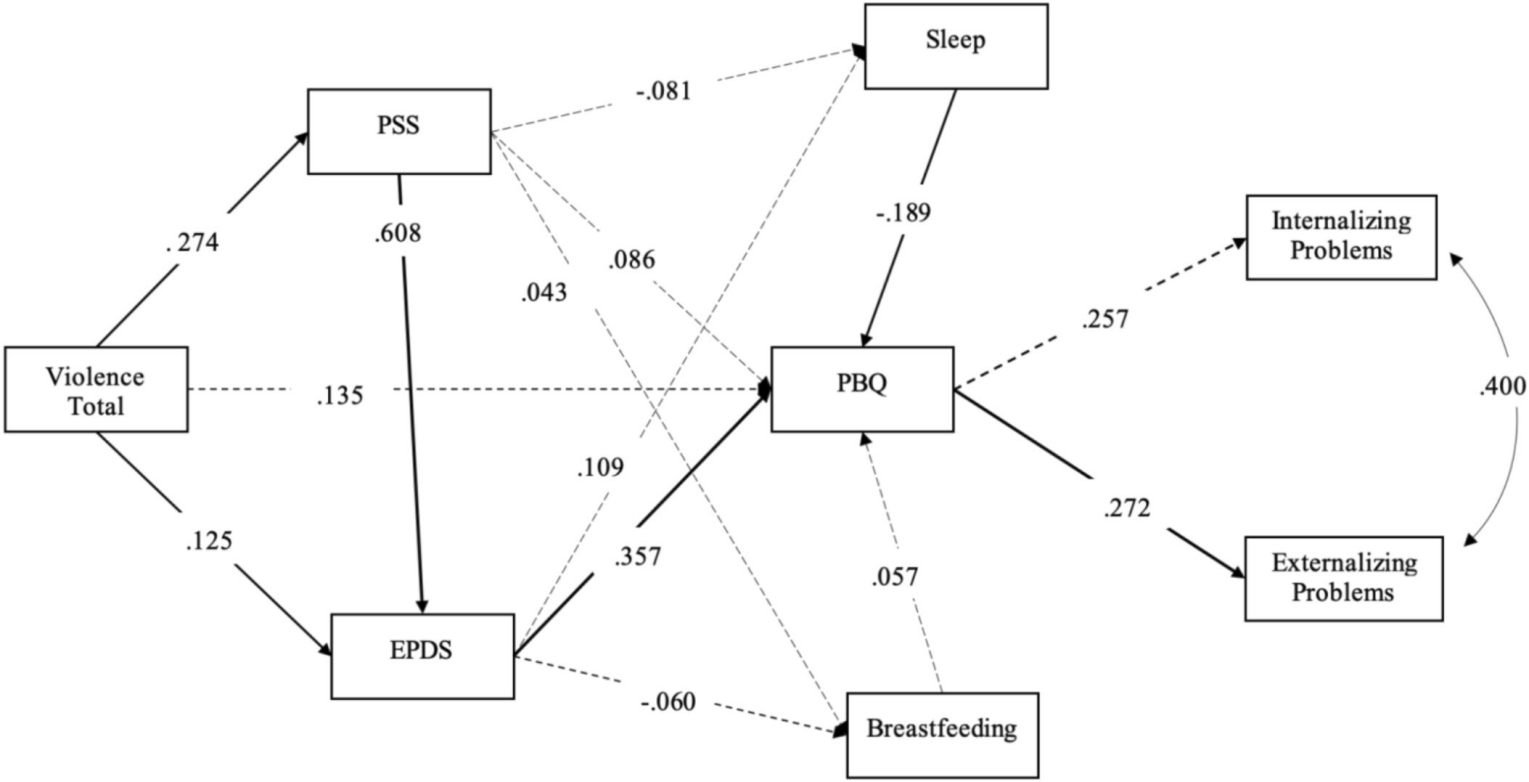
Complete *model* of *path analysis* to identify the associations between VT and mother–child bond failure, and the link in child's behaviour mediated by stress and postpartum maternal depression, infant sleep and breastfeeding (n. 294). Legend: Violence measured by a modified version of the Abuse Assessment Screen; Stress measured by the Perceived Stress Scale (PSS 14); postpartum depression measured by the Edinburgh Postnatal Depression Scale (EPDS); infant sleep through two questions taken from the Sleep Behaviour Questionnaire (SBQ); breastfeeding through a food frequency questionnaire; mother–baby bond failure measured by subscale 1 (weakened bond) of the Postpartum Bonding Instrument (PBQ); and the screening of child mental health problems by the Strength and Difficulties Questionnaire (SDQ) scale. The significant paths are in bold.

**FIGURE 3:**
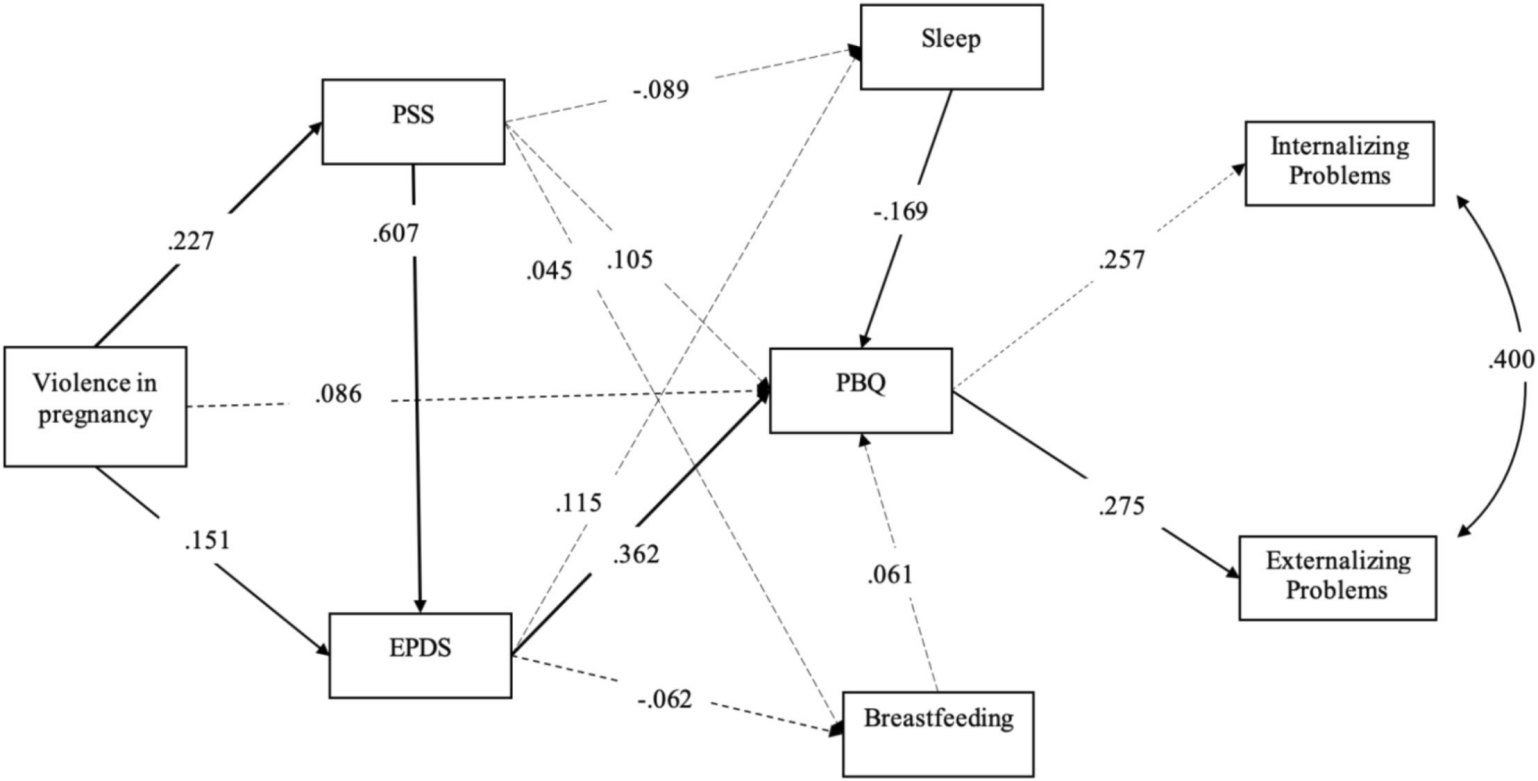
Complete *model* of *path analysis* to identify the associations between VP and mother–child bond failure, and the link in child's behaviour mediated by stress and postpartum maternal depression, infant sleep and breastfeeding (n. 294). Legend: Violence measured by a modified version of the Abuse Assessment Screen; Stress measured by the Perceived Stress Scale (PSS 14); postpartum depression measured by the Edinburgh Postnatal Depression Scale (EPDS); infant sleep through two questions taken from the Sleep Behaviour Questionnaire (SBQ); breastfeeding through a food frequency questionnaire; mother–baby bond failure measured by subscale 1 (weakened bond) of the Postpartum Bonding Instrument (PBQ); and the screening of child mental health problems by the Strength and Difficulties Questionnaire (SDQ) scale. The significant paths are in bold.

The exploratory model tested in the *path analysis* verified how much experiencing violent situations explained stress and depressive symptoms for the mother, and how much of these variables explained the amount of sleeping hours for the baby, breastfeeding and mother–child bond at 6 months. Finally, it was tested how much the mother–child bond influenced the presence of internalization or externalization problems for preschoolers. Although the SDQ instrument includes the prosocial behaviour dimension, the internal consistency of this subscale was low, compromising its reliability. In addition, the inclusion of Prosocial Behaviour in the structural model resulted in poor fit (CFI < 0.90; RMSEA > 0.08), indicating that the model was neither parsimonious nor theoretically adequate. For these reasons, we chose to report the results considering only internalizing and externalizing problems as outcomes, as it showed better fit and greater theoretical consistency. So, the results of this analysis, according to situations of violence in the mother's life, are represented in Figure 2 (CFI = 0.960; TLI = 0.914; RMSEA = 0.051; 90% CI 0.009–0.084; χ^2^(13) = 22.680, *p* = 0.05; SRMR = 0.077) and during pregnancy in Figure 3 (CFI = 0.986; TLI = 0.970; RMSEA = 0.029; 90% CI 0.000–0.068; χ^2^(28) = 228.959, *p* < 0.001; SRMR = 0.070). All reported coefficients represent standardized estimates. Standardized and unstandardized path coefficients, along with bootstrapped 95% confidence intervals, are presented in Table 3.

**TABLE 3:** Direct effects of total violence and violence during pregnancy on maternal and child outcomes with bootstrapped 95% confidence intervals.

Violence total							
Predictor		Outcome	B	SE	Lower 95% CI	Upper 95% CI	*β*
Violence total	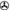	EPDS	1.242	0.529	0.174	2.291	0.125
Violence total	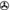	PSS	4.577	0.956	2.669	6.341	0.274
Violence total	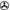	PBQ	1.160	0.678	−0.191	2.421	0.135
PSS	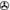	EPDS	0.362	0.038	0.287	0.433	0.608
PSS	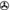	PBQ	0.045	0.048	−0.054	0.135	0.086
PSS	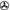	Breastfeeding	0.002	0.006	−0.010	0.012	0.043
PSS	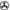	Sleep	−0.022	0.027	−0.074	0.028	−0.081
EPDS	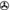	PBQ	0.310	0.104	0.135	0.547	0.357
EPDS	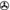	Breastfeeding	−0.006	0.010	−0.022	0.017	−0.060
EPDS	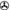	Sleep	0.050	0.046	−0.044	0.142	0.109
Sleep	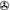	PBQ	−0.358	0.120	−0.573	−0.107	−0.189
LM6M	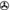	PBQ	0.536	0.583	−0.678	1.626	0.057
PBQ	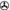	Internalizing problems	0.219	0.135	0.059	0.561	0.257
PBQ	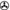	Externalizing problems	0.275	0.110	0.105	0.552	0.272

Violence during pregnancy							
Predictor		Outcome	B	SE	Lower 95% CI	Upper 95% CI	*β*
Violence during pregnancy	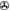	EPDS	2.053	0.903	0.220	3.888	0.151
Violence during pregnancy	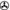	PSS	5.163	1.511	2.150	8.185	0.227
Violence during pregnancy	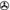	PBQ	1.017	0.865	−0.618	2.731	0.086
PSS	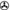	EPDS	0.362	0.035	0.293	0.432	0.607
PSS	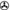	PBQ	0.054	0.046	−0.043	0.144	0.105
PSS	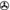	Breastfeeding	0.002	0.006	−0.010	0.012	0.045
PSS	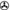	Sleep	−0.024	0.027	−0.076	0.027	−0.089
EPDS	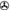	PBQ	0.314	0.104	0.139	0.543	0.362
EPDS	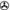	Breastfeeding	−0.006	0.010	−0.022	0.017	−0.062
EPDS	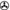	Sleep	0.053	0.046	−0.040	0.143	0.115
Sleep	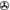	PBQ	−0.319	0.122	−0.544	−0.070	−0.169
LM6M	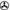	PBQ	0.567	0.582	−0.614	1.658	0.061
PBQ	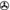	Internalizing problems	0.219	0.135	0.059	0.562	0.257
PBQ	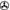	Externalizing problems	0.278	0.110	0.107	0.558	0.275

These models are rooted in well‐established theoretical frameworks that enlighten the relationships among the variables. Additionally, it is common that longitudinal studies are often affected by sample attrition, which may have limited the data statistical power of the analysis. Nonetheless, it is crucial to recognize that this study is primarily exploratory, aimed at unravelling complex relationships, which means that the results should not be interpreted as confirmatory evidence of hypothesized relationships. Therefore, the results offer valuable insights and serve as the grounds for further investigations.

The results showed that violence positively explained stress (see Figures 2 and 3). The statistically significant paths showed that stress positively explained depression; depression positively explained the weakened mother–child bond; the mother–child bond positively explained externalizing problems of preschoolers. As additional results, more hours of sleep were negatively associated with higher PBQ scores, which indicate a weaker mother–child bond. Therefore, the more hours of sleep, the stronger the mother–child bond. Also, breastfeeding was not associated either with maternal stress, PPD or the mother–child bond. In summary, the relation between violence and the mother–child bond was indirectly significant, mediated by stress and postpartum depression, and the weakened mother–child bond explained externalization problems of preschoolers.

### 3.2 Model Revision Rationale

In order to simplify the model and render the expected good fit, the prosocial behaviour dimension was excluded from subsequent analyses, driven by the poor consistency of the SDQ Prosocial subscale. We acknowledge a minor risk of non‐random missingness; however, this impact is considered negligible, as the excluded sample size was small and did not show systematic differences on key sociodemographic variables.

## 4 Discussion

In the search to provide evidence of the relation between the violence experienced by women and the mother–child bond and on the child's mental health at preschool age, it was possible to observe the correlation of violence with stress, and stress with postpartum depression; thus, showing that violence and the mother–child bond are indirectly significant, indicating the cycle of factors associated with the quality of the mother–child bond, and this bond on the child's mental health at preschool age, since the weakened bond is predictive of the development of externalization problems for the child.

### 4.1 Prevalence

The prevalence of violence was high: Approximately one out of two women was a victim of VT, and for VP, the prevalence was 15.9%. Similar results were found in a study conducted in Southeastern Brazil, in which 45.4% of the interviewees portrayed a history of violence by their partner throughout their lives, and nearly 12.0% were victims of violence during pregnancy (Santos et al. 2021). In the past 2 years, violence against women has significantly increased during the COVID‐19 pandemic (Bo et al. 2021; Everton Lima 2021). In Brazil, it is estimated that at every minute, eight women suffer some kind of physical violence (Brazilian Forum on Public Security 2022). The global prevalence of physical violence during pregnancy presents a wide variation (1.6%–78%), as well as psychological violence (1.8%–67.4%) (Román‐Gálvez et al. 2021). Violence against women is preventable (WHO 2021a), and therefore, interventions in order to minimize their prevalence and consequences must be carried out in a broad and systemic way, not only performed by health services, where the diagnosis of violence suffered by the victims is often late (Murray et al. 2020).

### 4.2 Violence and Socioeconomic Aspects

Most of the women who experienced violence in our sample were part of lower socioeconomic classes. VP was associated with lower maternal schooling, extremes of paternal schooling and absence of a partner. Among women who are assaulted, low schooling and lower income are common, which makes them more vulnerable to situations in which their rights are violated (Biswas et al. 2017). Regarding the assailants' profile, a survey conducted in Brazil identified young adults, married men with low education and paid work as characteristics (Scott and de Oliveira 2018).

### 4.3 Outcomes of Violence and Mother–Child Health

The violence experienced by women can also be very harmful to the child's health. In our study, the children of women who were victims of violence during pregnancy were born with lower birthweight and lower head circumference. These data are in line with the results of other studies (Garg et al. 2020; Luhumyo et al. 2020). At preschool age, children of women victims of VT and VP presented higher scores for externalization problems. A systematic review by Silva et al. (2018) identified twice as higher the chance for internalization problems and 1.90% for externalization problems in children of women exposed to violence during pregnancy when compared to children of those who were not exposed to violence.

Economic stress and lower education should also be evaluated and monitored to avoid postpartum depression (Edwards et al. 2021), as well as mental health problems in children, which may also be influenced by family income (Collishaw et al. 2019). However, even those women with less purchasing power may have social support, as an important mediator for the mother–child bond, considering that the neighbourhood can moderate the effects of intimate partner violence on the bonds with the child (Martinez‐Torteya et al. 2021).

Violence is recognized as a significant predictor of maternal mental health problems (Dadi et al. 2020; Edwards et al. 2021; Paulson 2022). In a survey conducted in Cameroon, the accumulation of child maltreatment and the intimate partner violence experienced by the mother was associated with symptoms of anxiety and depression of the mother, as well as symptoms of externalization problems for the children (Wadji et al. 2022). Women who experienced verbal violence during pregnancy were more likely to physically abuse their 4‐month‐old child, and, after adjusting for the depression score and the mother–child bond, the association remained significant, suggesting the influence of verbal abuse from an intimate partner on the child (Amemiya and Fujiwara 2016). Park et al. (2021) observed that emotional and physical violence were associated with symptoms of depression, which is related to a weak mother–child bond, whereas, in isolation, emotional violence had also a direct effect on the mother–child bond (Park et al. 2021).

In Poland, Lutkiewicz et al. (2020) investigated the relation between the mother–child bond and maternal symptoms of stress, depression and anxiety in the postpartum period, and they found out that the higher the maternal depression and stress scores, the more problematic the mother–child bond was (Lutkiewicz et al. 2020). Children raised by depressed mothers are more likely to present fear and anxiety (Slomian et al. 2019). Internalization problems were identified in children of adolescent mothers who had a diagnosis of major depression during the child's first year of life (Bagner et al. 2010).

Different experiences of violence, sexual, emotional, physical abuse or domestic violence or even any kind of violence against the child are significantly associated with a higher risk of developing postpartum depression (Zhang et al. 2019). Postpartum depression increased from 18.2% to 25.6% in low‐ and middle‐income countries between 2010 and 2017. A review of 58 studies by Dadi et al. (2020) found a wide variation in the incidence of postpartum depression, ranging from 3.5% in Ghana to 58.8% in Iran, while the effect on the child's health was 31%. Another study investigating data from 80 countries found a global prevalence of postpartum depression, being the highest in South Africa (39%) and also in developing countries (Wang et al. 2021). The strongest predictors for postpartum depression are the support received from the partner/father (Edwards et al. 2021), intimate partner violence, particularly from the current partner, and prenatal depression (Hutchens and Kearney 2020). There was also an increase in mental health problems in pregnant and puerperium women during the COVID‐19 pandemic, and the prevalence of postpartum depression during that period as well (Bo et al. 2021; Safi‐Keykaleh et al. 2022).

Many studies negatively associate intimate partner violence and postpartum depression with exclusive and non‐exclusive breastfeeding (Normann et al. 2020). However, in the present study, breastfeeding was not explained by either depression or stress, nor did it explain the quality of the mother–child bond. In our cohort, breastfeeding did not show significant associations with other variables, which may indicate that this variable was not well measured or collected (Souza et al. 2024).

Our results also corroborate with those of other studies that investigated the association of violence with the mother–child bond and mental health in childhood. The study by Kita et al. (2016) found an association between intimate partner violence during pregnancy and failure on the mother–child bond 1 month after the childbirth, yet no association between postpartum violence and depression was found in the same period (Kita et al. 2016).

### 4.4 Strengths and Limitations

Among the strengths of this study, it is important to highlight its longitudinal design including retaining a considerable number of mothers after 5 years of follow‐up. This design allows for a better understanding of temporal relationships between exposure to violence and developmental outcomes, thus strengthening causal inferences. The frequency of follow‐up interviews minimizes recall and memory bias, ensuring more accurate and reliable data over time. Furthermore, the study's comprehensive approach—integrating the investigation of violence with a broad range of maternal, child and contextual variables—enables a more nuanced and multidimensional analysis of associations, contributing to a deeper understanding of the complex mechanisms underlying child development.

Within the limitations of this study, here, it is presented a homogeneous profile sample of women regarding social class, age, marital status and partner's work situation, restraining comparisons with other studies presenting a different population sample. In addition, we had to exclude subjects with missing data in the variable ‘violence’, which can be considered a limitation as the presence of non‐random missing data may limit the generalizability of the results.

The prevalence of violence may have been underreported, as many women are unaware of the clear definition of violence, as questionnaires were self‐reported, without prior conceptual clarification on the subject. The questionnaire used to measure violence may not have fully captured the complexity of this multifaceted issue. Another important reason for the underreporting was the profile of women who answered the questionnaire on violence, mainly because they were older when compared to the group that did not answer it.

Likewise, no information on depressive disorder symptoms was collected in the prenatal period, which would better identify the profile of women with a predisposition to violence. Thus, the suboptimal fit indexes in our path analysis should be interpreted within the context of these limitations, and the results should be considered under the awareness of these methodological challenges; nevertheless, our findings are consistent with the existing literature.

Regarding the behaviour of children, one limitation of this study lies in using heterogeneous reports to evaluate children's characteristics, as caregivers provided all responses. Another limitation is that we did not ask mothers whether they were experiencing violence in the present, which could be affecting the child's behaviour. However, it is undeniable that the violence experienced by the mother during her lifetime or during pregnancy also affects her depressive symptoms, which in turn influence the level of bonding she has with the child and, consequently, the child's externalizing problems.

### 4.5 Clinical, Research, Political and Social Implications

The results of the present study have important implications for clinical and research practice, since they may help to develop prevention and care strategies, as they reinforce the evidence that violence, maternal mental health, mother–child bond and child's health are linked.

#### 4.5.1 For Mothers

Currently, many of the maternal and child health care programmes in the postpartum period have the newborn as their main focus. However, attention to the mother's psychological aspects must be strengthened by contributing to trace maternal mental health problems, avoiding future problems for the mother–child pair and higher social and economic costs.

Information, guidance, prevention and health promotion at school level, for example, can be accessible to younger women who are the most exposed to violence (USP/AUN 2019). Postpartum depression, identified as a mediating factor between violence and mother–child bond, also affects women in the lower age groups, with a history of previous depression, low family support and low economic power, thus interfering in the structure of maternal behaviour and in the bond with the child (Hakanen et al. 2019; Hymas and Girard 2019; Tichelman et al. 2019).

Psychological interventions have already been studied for this context and have also been effective in the treatment of perinatal depression (Cuijpers et al. 2021; Gajaria and Ravindran 2018; Huang et al. 2020). For example, psychological prenatal care, complementary to traditional prenatal care, is aware of the subjectivities during pregnancy, and it constitutes a psychoprophylactic instrument, which must be implemented as a public health policy (Almeida and da R. 2016).

#### 4.5.2 For Mother–Baby

There are recent studies indicating favourable results of parental and child interaction therapies (Kingsley et al. 2020; Tien et al. 2020). The so‐called psychoeducation and training of facial expression recognition can also be effective for the treatment and recovery of depressed women, contributing to creating a bond with their children (Letourneau et al. 2017). Skin‐to‐skin contact can regulate stress, anxiety and psychological distress for the mother and the baby, and has proved to be an effective and low‐cost therapy (Ionio et al. 2021; Kirca and Adibelli 2021). In public health care, spaces of guidance and fostering for mother–child pairs strengthen changing processes (Peres and dos Santos 2018).

#### 4.5.3 For the Children

The violence suffered by the mother interferes in the parental education, with a high prevalence of violent maternal educational practices (Silva et al. 2017). Evaluating the global development of children considering their psychological aspects is necessary, with special attention to the daughters of younger women, who are victims of violence, with low income and mental health problems, in order to identify vulnerabilities and to promote care and support for the victims' children.

## 5 Conclusions

One of the major findings of our study indicates that there is not only one, but a few critical points in which one must take measures to prevent mental health problems in children. These findings show the importance of identifying women prone to violence and investing in preventive programmes. Besides evaluating the quality of the maternal‐fetal and the mother–child bond in prenatal care and the child's welfare, some measures may be implemented during these periods, such as identifying vulnerabilities, knowing the level of perceived stress and the occurrence of prenatal and postpartum depression and using accessible and low‐cost community services.

Breaking the cycle of violence against women, postpartum stress and depression, weakening the mother–child bond, thus ending in the infant's behavioural problems, must be urgent, besides observing the possible transgenerational character of depressive symptoms and the importance of investing in programmes to help women at a very early age in the construction of the mother–child bond.

## Author Contributions

**Viviane Costa de Souza Buriol:** conceptualization, methodology, validation, investigation, writing – original draft, visualization. **Marina Nunes:** formal analysis, investigation, data curation. **Ariela Raissa Lima‐Costa:** formal analysis, data curation. **Marcelo Zubaran Goldani:** resources, writing – review and editing, supervision, funding acquisition. **Juliana Rombaldi Bernardi:** investigation, supervision. **Denise Ruschel Bandeira:** writing – review and editing, supervision, project administration. **Clécio Homrich da Silva:** writing – review and editing, supervision, project administration.

## Funding

Financial support for research was provided by the Program of Support to Center of Excellence in Science, Technology & Innovation of the Conselho Nacional de Desenvolvimento Científico e Tecnológico (PRONEX/CNPq—2009); the Fundação de Amparo à Pesquisa do Estado do Rio Grande do Sul/Conselho Nacional de Desenvolvimento Científico e Tecnológico (FAPERGS/CNPq—10/0018.3) and the Research and Events Incentive Fund of the Hospital de Clínicas de Porto Alegre (HCPA).

## Ethics Statement

The Ethics and Research Committee of Hospital de Clínicas de Porto Alegre (HCPA) (Protocol 11/0097) and the Grupo Hospitalar Conceição (GHC) (Protocol 11/027) approved the IVAPSA Cohort and, therefore, the present study. All participants were recruited after signing the Informed Consent Form. The project was carried out in accordance with the latest guidelines and current norms of the National Health Council of the Brazilian Ministry of Health (resolutions numbers 466/2012 and 580/2018). During the interviews, when the health conditions of the participants were an object of concern, they were referred to the reference services.

## Conflicts of Interest

The authors declare no conflicts of interest.

## Data Availability

The data that support the findings of this study are available from the corresponding author upon reasonable request.
